# High growth hormone serum partially protects mice against *Trypanosoma cruzi* infection

**DOI:** 10.1002/2211-5463.13627

**Published:** 2023-05-15

**Authors:** Patricia Mora‐Criollo, Reetobrata Basu, Yanrong Qian, Kevin Funk, Stephen Bell, Jonathan A. Young, Edward O. List, Jaime A. Costales, Jaime Guevara‐Aguirre, Mario J. Grijalva, John J. Kopchick

**Affiliations:** ^1^ Department of Biomedical Sciences, Infectious and Tropical Disease Institute Heritage College of Osteopathic Medicine, Ohio University Athens OH USA; ^2^ Edison Biotechnology Institute Ohio University Athens OH USA; ^3^ Heritage College of Osteopathic Medicine Ohio University Athens OH USA; ^4^ Centro de Investigación para la Salud en América Latina, Escuela de Ciencias Biológicas, Facultad de Ciencias Exactas y Naturales Pontificia Universidad Católica del Ecuador Quito Ecuador; ^5^ Colegio de Ciencias de la Salud Universidad San Francisco de Quito Ecuador; ^6^ Faculty of Health Medicine and Life Sciences Maastricht University The Netherlands

**Keywords:** bGH, Chagas disease, GHR^−^^/^^−^ mice, growth hormone, Laron syndrome, *Trypanosoma cruzi*

## Abstract

Chagas disease (CD) is one of the most devasting parasitic diseases in the Americas, affecting 7–8 million people worldwide. *In vitro* and *in vivo* experiments have demonstrated that growth hormone (GH) serum levels decrease as CD progresses. Interestingly, inactivating mutations in the GH receptor in humans result in Laron syndrome (LS), a clinical entity characterized by increased serum levels of GH and decreased insulin growth factor‐1 (IGF‐1). The largest cohort of LS subjects lives in the southern provinces of Ecuador. Remarkably, no clinical CD cases have been reported in these individuals despite living in highly endemic areas. In the current *ex vivo* study, we employed serum from GHR^−/−^ mice, also known as LS mice (a model of GH resistance with high GH and low IGF‐1 levels), and serum from bovine GH (bGH) transgenic mice (high GH and IGF‐1), to test the effect on *Trypanosoma cruzi* infection. We infected mouse fibroblast L‐cells with *T. cruzi* (etiological CD infectious agent) and treated them with serum from each mouse type. Treatment with GHR^−/−^ serum (LS mice) significantly decreased L‐cell infection by 28% compared with 48% from control wild‐type mouse serum (WT). Treatment with bGH mouse serum significantly decreased infection of cells by 41% compared with 54% from WT controls. Our results suggest that high GH and low IGF‐1 in blood circulation, as typically seen in LS individuals, confer partial protection against *T. cruzi* infection. This study is the first to report decreased *T. cruzi* infection using serum collected from two modified mouse lines with altered GH action (GHR^−/−^ and bGH).

## Graphical abstract

High growth hormone (GH) serum collected from GHR^−/−^ mice, also called Laron mice (LS) [high GH and low insulin growth factor‐1 (IGF‐1) levels] and transgenic bovine GH mice (high GH and low IGF‐1 levels), significantly decreased *Trypanosoma cruzi* infection in fibroblast L‐cells. Altered GH action (high GH) seems to play a protective role during *T. cruzi* infection, the parasite responsible for Chagas disease.

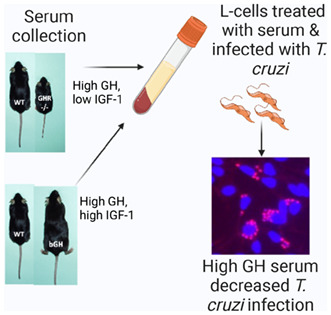

**Abbreviations**
AC: acromegaly
bGH: bovine growth hormone mice
BRC: bromocriptine
CD: Chagas disease
DAPI: 4′,6‐diamidino‐2‐phenylindole
DMEM: Dulbecco's modified Eagle's medium
DMEM10: Dulbecco's modified Eagle's medium with 10% FBS
DMEM2: Dulbecco's modified Eagle's medium with 2% FBS
ELISA: enzyme‐linked immunosorbent assay
GC: glucocorticoid
G‐CSF: granulocyte colony‐stimulating factor
GH: growth hormone
GHD: growth hormone deficiency
GHR: growth hormone receptor
GHR^−^^/^^−^
: growth hormone receptor knock‐out mice
GHR^+^^/^^−^
: heterozygote for GHR
GHR^+^^/^^+^
: homozygote for GHR
GTT: glucose tolerance test
HPA: hypothalamic–pituitary–adrenal axis
hpi: hours postinfection
IFN‐γ: interferon‐gamma
IGF‐1: insulin growth factor‐1
IGHD: isolated growth hormone deficiency
IL‐12: interleukin‐12
IL‐13: interleukin‐13
IL‐1‐α: interleukin‐1‐alpha
IL‐1‐β: interleukin‐1‐beta
ITT: insulin tolerance test
LS mice: Laron syndrome mice
LS: Laron syndrome
MET: metoclopramide
mIGF‐1: mouse IGF‐1
MT: metacyclic trypomastigotes
NO: nitric oxide
PBS: phosphate‐buffered saline
PI: postinfection
PRL: prolactin
*T. cruzi*
: *Trypanosoma cruzi*
TNF‐α: tumor necrosis factor‐alpha
WT: wild‐type mice

Chagas disease (CD) is a parasitic disease caused by the protozoan parasite *Trypanosoma cruzi*. Approximately 7–8 million people are currently infected worldwide, leading to ~ 50 000 deaths per year [1]. Of those infected, 5 million are found in South American countries. Due to migration and globalization in recent decades, CD has spread globally to nonendemic areas such as Canada, the USA, Europe, Australia, and Japan [2]. Transmission of CD in endemic areas occurs mainly through contact with contaminated feces of triatomine insects, also known as kissing bugs [3]. Less frequent infection routes include oral transmission, contaminated food, or vertical transmission from mother to child during pregnancy and childbirth [3, 4, 5]. Clinical manifestations of CD infection involve an initial acute stage with high parasitemia and display no or mild symptoms such as fever and anorexia [6]. Subsequently, CD progresses to a chronic phase that may present with clinical abnormalities such as cardiomyopathy or nervous system abnormalities that can cause incapacity and even death [5, 7]. Therapy for the acute phase of CD is limited to two oral antiparasitic drugs commercially known as nifurtimox and benznidazole [8]. Unfortunately, there is no effective treatment for the chronic stage of CD [7, 9].

Growth hormone (GH) is a protein secreted from the anterior pituitary gland that regulates postnatal growth, metabolism, and organ development [10]. GH production and secretion are regulated by hypothalamic GH‐releasing hormone, somatostatin, stomach‐derived ghrelin, and endocrine insulin growth factor‐1 (IGF‐1) [11]. Changes in GH activity have been associated with various diseases in humans. For instance, untreated oversecretion of GH by pituitary adenoma results in acromegaly (AC) in adults and gigantism in children. AC is a slowly progressive disease caused by chronic hypersecretion of GH with a concomitant increase in circulating IGF‐1 produced primarily by the liver [11]. By contrast, decreased secretion of GH results in GH deficiency (GHD) and is associated with impeded growth and other abnormalities in children. An extreme condition known as Laron syndrome (LS) is caused by homozygous inactivating mutations in the growth hormone receptor (GHR) gene (GHR^−/−^) and is characterized by GH insensitivity [12]. LS subjects are resistant to GH and have decreased serum levels of IGF‐1 and elevated GH levels, have severely diminished stature, and are obese. In an apparent paradox, these subjects display enhanced insulin sensitivity due to the absence of the GH counter‐regulatory effects on carbohydrate metabolism and a diminished incidence of cancer and diabetes [13, 14]. LS subjects also display slower cognitive decline than their age and sex‐matched relatives (GHR^+/+^ or GHR^+/−^) [13, 15]. The largest cohort of LS subjects live in the southern provinces of Ecuador [13, 16], and despite living in highly CD endemic areas, no clinical cases of this parasitic infection have been reported (Jaime Guevara‐Aguirre, personal communication). Interestingly, the absence of CD in LS patients from Ecuador resonates with a large cohort of adult GH deficiency patients (GHD‐decreased IGF‐1 serum levels) from Brazil, where no cases of CD were observed [17].

Emerging evidence suggests that GH influences the progression of *T. cruzi* infection [18, 19, 20] (Table 1). Moreover, *T. cruzi* infection directly promotes decreased GH and prolactin (PRL) production by the pituitary [18]. Notably, GH and PRL are known to inhibit parasitic infections by enhancing the immune response in the host by increasing the concentrations of tumor necrosis factor‐alpha (TNF‐α), interleukin 12 (IL‐12), interferon‐gamma (IFN‐γ), and nitric oxide (NO) production [17, 20, 21, 22]. For example, rats infected with *T. cruzi* and treated with GH resulted in decreased parasitemia in the blood leading to an improved immune response (increased TNF‐α, NO, and IFN‐γ) compared with nontreated controls [23]. Accordingly, our previous *in vitro* studies showed that human HeLa and mouse fibroblast L‐cells infected with *T. cruzi* and treated with relatively high GH concentrations have significantly less CD infection [24]. Moreover, the combination of high GH and low IGF‐1 levels, simulating LS conditions *in vitro*, decreased *T. cruzi* infection by preventing parasitic cell invasion into the cells [24]. When human HeLa cells were treated with a GH receptor antagonist (Pegvisomant), the levels of infection were restored similarly to the control levels (PBS) [24]. These data strongly suggest that GH influences *T. cruzi* infection *in vitro*.

**Table 1:** Effect of GH, PRL, and IGF‐1 during *Trypanosoma cruzi* infection. BRC, bromocriptine; GTT, glucose tolerance test; IGHD, isolated growth hormone deficiency; ITT, insulin tolerance test; MET, metoclopramide.

Model	Parasite/strain	Treatment	Results	Mechanisms	Ref.
Patients with CD chronic phase of infection	*T. cruzi*	–	Decreased GH levels in response to GTT and ITT compared with healthy subjects	Not explained	[45]
Rat pituitary GH3 cells	*T. cruzi*	–	Reduced GH secretion and PRL levels by the parasite	*T. cruzi* infection downregulated GH‐PRL production by the pituitary	[18]
Wistar Rats	*T. cruzi* (Y strain)	GH (5 ng/10 g body weight)	GH reduced trypomastigotes burden in blood and tissue	Increased NO, TNF‐α, IFN‐γ production	[23]
Wistar Rats	*T. cruzi* (Y strain)	PRL (40 μg·day)	PRL reduced trypomastigotes in blood	Depletion of T lymphocytes CD4^+^ CD8^+^, PRL increased IFN‐γ and NO production	[46]
Male BALB/c mice	*T. cruzi* (Tulahuen)	PRL antagonism (BRC 10 mg·kg per 100 μL) and agonist (MET 2.5 mg·kg per 100 μL)	BRC decreased PRL, increased GC levels, and induced thymic atrophy. MET increased PRL and protected against thymic atrophy	Depletion of CD4^+^ CD8^+^ T cells by induced apoptosis. Increased CD4^+^ CD8^+^ T cells	[19, 47]
Isolated macrophages from IGHD patients	*Leishmania amazonensis*	IGF‐1 (75 ng·mL)	Increased infection	Decreased NO, increased arginase activity	[44]
L‐cells HeLa cells	*T. cruzi* (Brazil)	Bovine GH + mouse IGF‐1 (200 ng·mL + 50 ng·mL) Human GH + human IGF‐1 (50 ng·mL + 20 ng·mL)	Decreased infection and less parasite entrance into the cells	Conversion of trypomastigotes into amastigotes resulted in less parasite invasion into the cell	[24]

In the current study, we used serum collected from LS GHR^−/−^ mice (elevated GH, decreased IGF‐1) and AC bGH mice (elevated GH, elevated IGF‐1) previously generated in our laboratory to assess the effect of on *T. cruzi* infection [25, 26]. Results showed that elevated GH and diminished IGF‐1 serum levels significantly protect against *T. cruzi* infection. This study is the first to explore the absence of *T. cruzi* infection in LS subjects using an *ex vivo* GH insensitivity mouse model. Our results suggest that serum from GHR^−/−^ mice confer partial protection against *T. cruzi* infection.

## Materials and methods

### Cell culture

Epithelial male mouse fibroblast cell line, strain C3H/AN (L‐cells) from ATCC^®^ were used in this study. This cell line was selected because it expresses GH receptors (GHR) [27]. Cells were cultured in Dulbecco's modified Eagle's medium (DMEM) (ATCC^®^ 30‐2002™, Manassas, VA, USA), supplemented with 10% FBS from ATCC^®^, 100 U·mL^−1^ penicillin–streptomycin (DMEM10) (Gibco™, Miami, FL, USA; catalog number 1600044) and maintained at 37 °C with a 5% CO_2_ atmosphere.

### Parasite maintenance

The parasite *T. cruzi* [strain Brazil (TcI)] life‐stage epimastigotes were cultured in liver‐infusion‐tryptose broth and supplemented with inactivated 10% FBS (Gibco™; catalog number 1600044). Inactivation of FBS serum complement components is essential for parasite survival and to ensure infection of cells. Epimastigotes were starved for 15 days until metacyclic trypomastigotes (MT) formed spontaneously and then subsequently used for infection. Commercial horse serum (Fisher Scientific, Waltham, MA, USA; catalog 35030CV) was implemented to eliminate remaining epimastigotes in the media [28]. MTs were then collected, washed in PBS 1×, suspended in DMEM supplemented with 2% FBS (DMEM2), and used to infect mouse L‐cells as previously described [24, 29]. After several rounds of replication, tissue‐derived trypomastigotes were collected 4–5 days postinfection (PI) and used for further L‐cells infection as described before [24].

### Mouse lines

We have previously generated two mouse models in our laboratory: GHR^−/−^ mice and bGH mice with a C57BL/6J genetic background [25, 26]. The GHR^−/−^, also known as the LS mouse, exhibits increased serum GH and decreased IGF‐1 and insulin concentrations [25]. Conversely, bovine GH transgenic (bGH) mice possess high GH and IGF‐1 serum levels, resembling the characteristics of untreated AC patients [30]. Serums from control wild‐type (WT) C57BL/6J mice, GHR^−/−^ mice, and bGH mice were used in our experiments.

### Serum collection

Serum was collected following the protocol approved by the Institutional Animal Care and Committee of Ohio University protocol #12H012. Sexually mature male mice at 3‐month‐old were used in all experiments [31]. Three‐month‐old male mice were selected because the C57BL/6J mice at this stage mice had reached sexual maturity and appeared fully developed as young adults [31]. Before bleeding, mice were fasted for 6 h, then the blood sample was obtained by cutting 1 mm from the tip of the mouse tail and was collected using microvette CB300 tubes (Fisher Scientific) [32]. Collected blood was kept on ice for 20 min (min) at room temperature (RT), then spun at 6000 ***g*** for 15 min to remove the clot. Six bGH (strain C57BL/6J) and six WT (strain C57BL/6J) mice littermate controls and six GHR^−/−^ (strain C57BL/6J) and six WT (strain C57BL/6J) mice littermate controls were bled every month for six consecutive months. Serum samples from each mouse were kept individually at −80 °C for 6 months, thawed, and pooled immediately before experiments. In total, six serum samples from each mouse were used for the infection experiments. Next, serum was aliquoted for measurement of GH and IGF‐1 levels via ELISA and simultaneously used for treating cells [32]. Serum glucose levels were determined using a glucose testing kit (Contour next strips, Contour next EZ^®^, Parsippany, NJ, USA).

### Treatment of cells

**Fig. 1:**
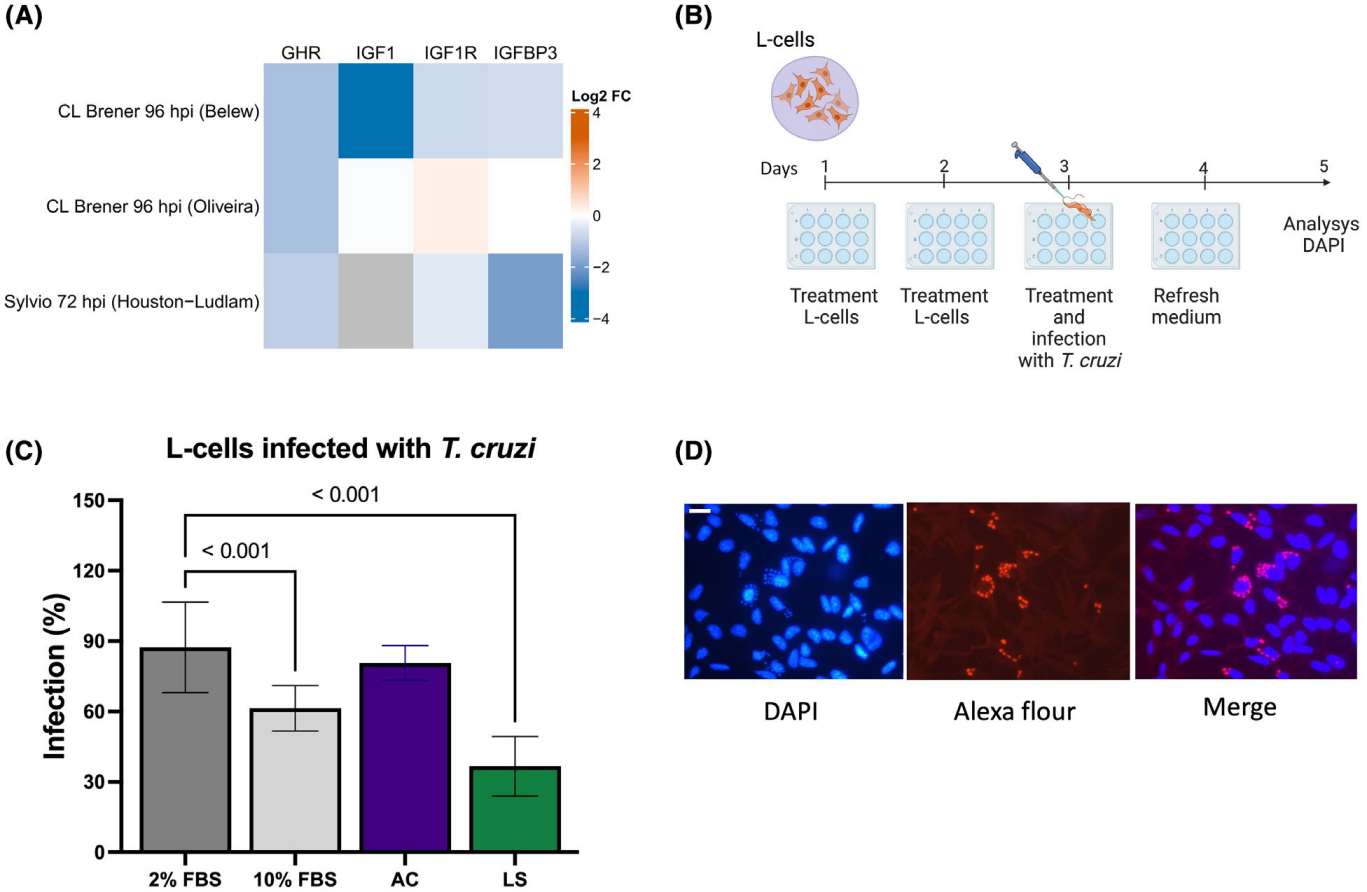
(A) Heat map of differentially expressed *GHR, IGF‐1, IGF‐1R*, and *IGFBP3* genes across *Trypanosoma cruzi* infected cells. The heat map was generated using previously generated RNA‐seq data [37, 38, 39] from human foreskin fibroblast infected with *T. cruzi* strain CL Brenner and strain Sylvio at 96 and 72 h postinfection (hpi), respectively. On the heap map, red meaning upregulated gene expression, and blue meaning downregulated gene expression. (B) Protocol used to infect and treat L‐cells. (C) L‐cells were treated with 2% FBS or 10% FBS or bGH (200 ng·mL) + mIGF‐1 (900 ng·mL) (AC conditions) or bGH (200 ng·mL) + mIGF‐1 (50 ng·mL) (LS conditions) followed by *T. cruzi* infection. Infection (%) was calculated. Two independent experiments with three replicates each were performed. Results are expressed as mean ± SD. A one‐way ANOVA followed by a *post‐hoc* Bonferroni's comparison test was performed. A *P*‐value of < 0.05 was considered statistically significant. (D) L‐cells fixed and incubated with secondary antibody Tc‐cyp19, Alexa‐fluor, that binds specifically to intracellular amastigotes followed by DAPI stained. The scale bar is 10 μm.

Recombinant bovine GH (bGH) (catalog # CYT‐636) and recombinant mouse IGF‐1 (mIGF‐1) (catalog # CYT‐229) were purchased from Prospec‐Tany Technogene (https://www.prospecbio.com). For the *in vitro* experiments, we used L‐cells cultures treated with DMEM2 or DME10 or bGH (200 ng·mL) + mIGF‐1 (900 ng·mL) simulating AC conditions or bGH (200 ng·mL) + mIGF‐1 (50 ng·mL) simulating LS conditions [24]. For treatment of L‐cells with (bGH + mIGF‐1), DMEM2 was added every 24 h for 4 days (day 1, 2, 3, 4), followed by infection with *T. cruzi* (day 3) for 24 h. Then, the cells were washed once with PBS (2 mL), and parasites were removed (day 4). L‐cells were infected with trypomastigotes using 1 × 10^6^ parasites per cell for 24 h, as previously described [24]. Infection of cells was analyzed at 48 h PI on day 5 (Fig. 1B–D).

**Fig. 2:**
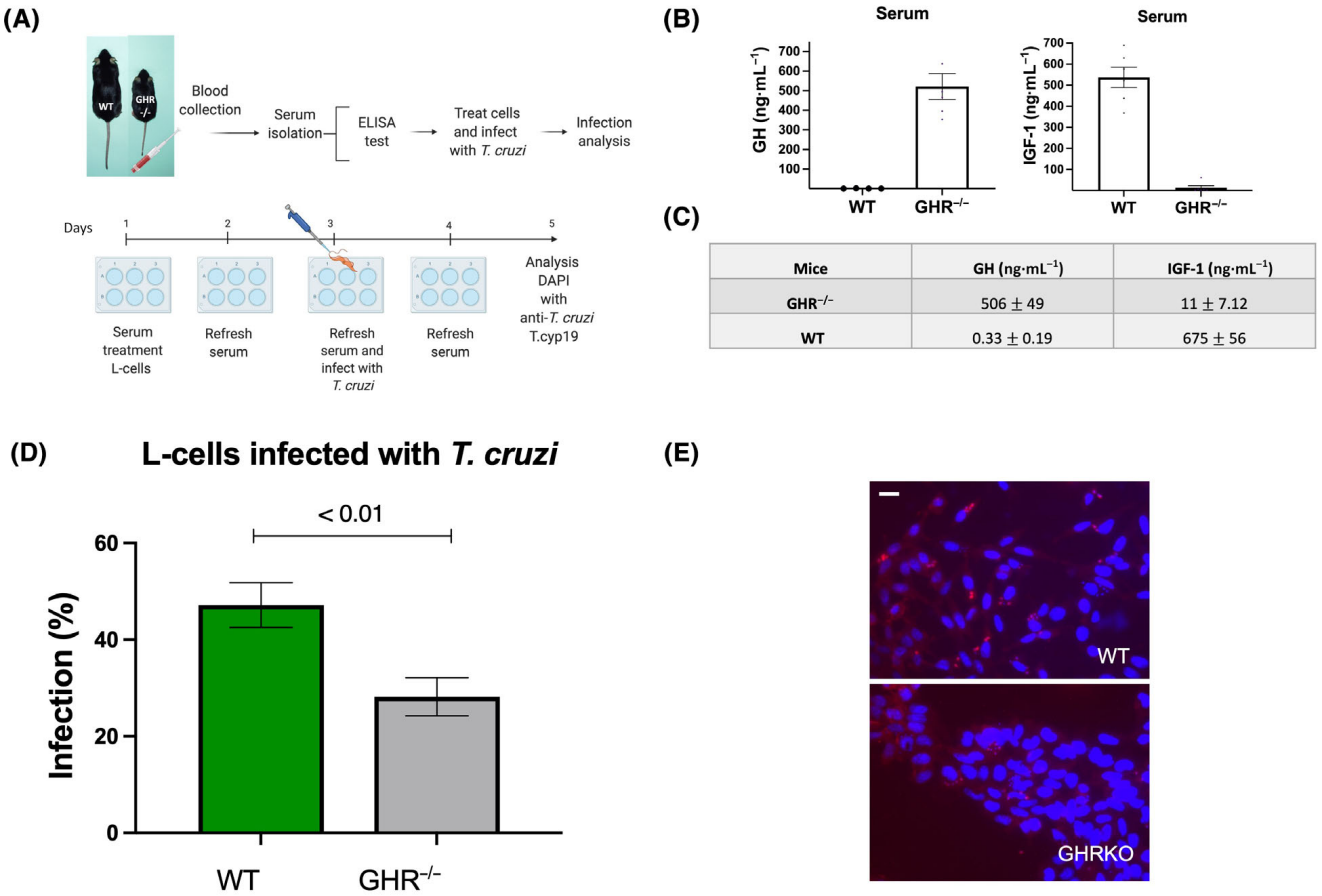
L‐cell treatment with mouse GHR^−/−^ serum followed by infection with *Trypanosoma cruzi*. (A) Protocol used for this experiment. (B, C) GH serum concentration from WT and GHR^−/−^ mice (*n* = 6). IGF‐1 serum concentration from WT and bGH mice (*n* = 6). (D) L‐cells were treated with WT (*n* = 6) and GHR^−/−^ mice serum (*n* = 6) and then infected with *T. cruzi*. Three sterile coverslips were placed on 12 well plates, and L‐cells were seeded on top. Infection (%) was calculated. Error bars represent SEM. An unpaired *t*‐test was performed in graphpad prism version 9.1.2. A *P*‐value of < 0.05 was considered statistically significant. (E) L‐cells fixed and incubated with secondary antibody Tc‐cyp19, Alexa‐fluor, and DAPI stained. The scale bar is 10 μm.

**Fig. 3:**
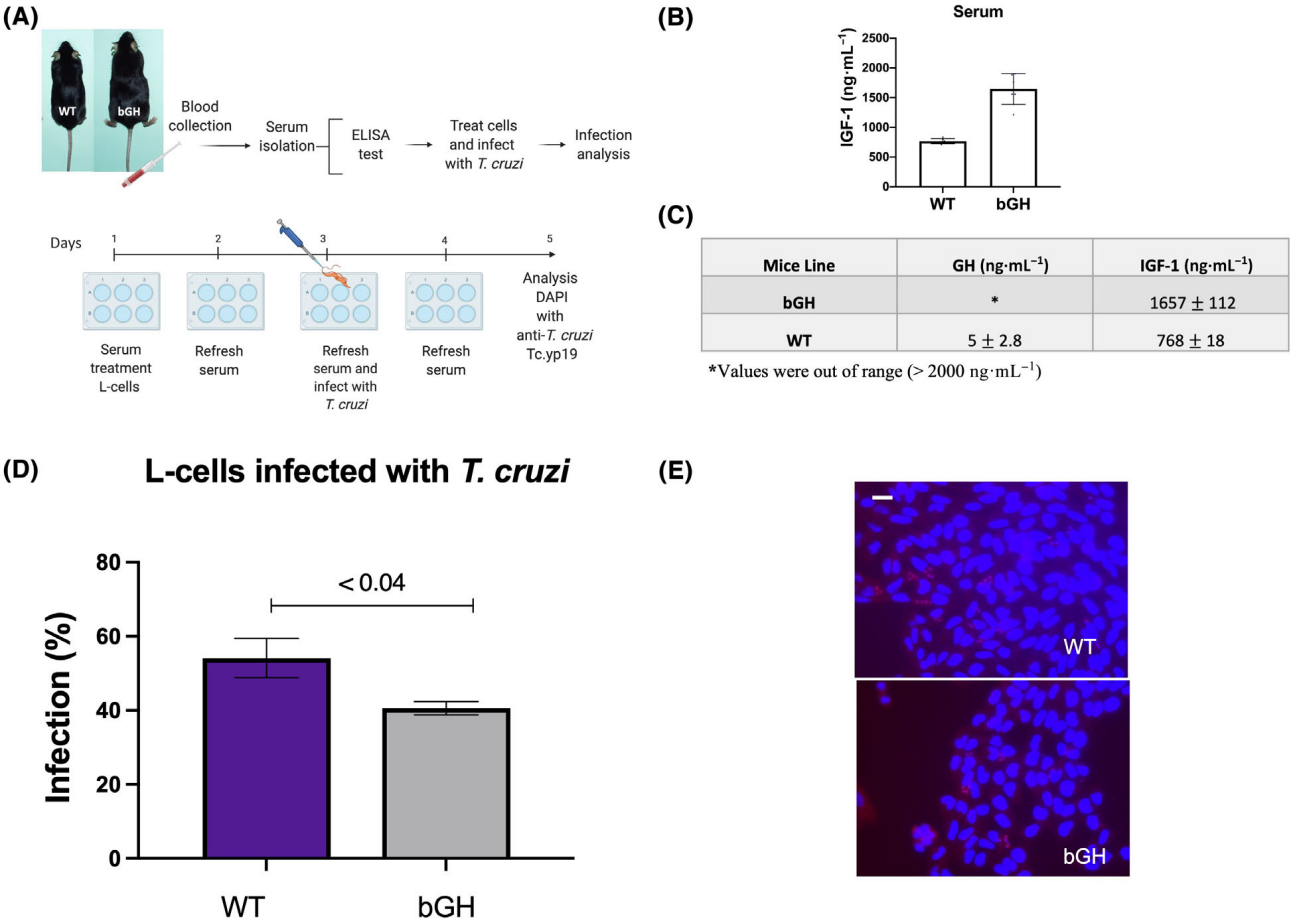
Treatment of L‐cells with bGH mice serum and infection with *Trypanosoma cruzi*. (A) Protocol used for this experiment. (B, C) IGF‐1 serum concentration from WT (*n* = 6) and bGH mice (*n* = 6). GH serum concentrations were above detectable values (> 2000 ng·mL). (D) L‐cells were treated with WT (*n* = 6) and bGH mice serum (*n* = 6) and then infected with *T. cruzi*. Three sterile coverslips were placed on 12 well plates, and L‐cells were seeded on top. Infection (%) was calculated. Error bars represent SEM. An unpaired *t*‐test was performed. A *P*‐value of < 0.05 was considered statistically significant. (E) L‐cells fixed and incubated with secondary antibody Tc‐cyp19, Alexa‐fluor, and DAPI stained. The scale bar is 10 μm.

For the *ex vivo* experiments (Figs 2 and 3), mouse L‐cells were treated with GHR^−/−^ mouse serum (10%) + DMEM or bGH mouse serum (10%) + DMEM for four consecutive days (days 1, 2, 3, 4), with serum added every 24 h, followed by infection with *T. cruzi* (day 3). Infection proceeded for 24 h using 1 × 10^6^ parasites per cell. After that time, cells were washed, and parasites were removed, as above. Infection of cells was analyzed at 48 h PI (day 5).

### Infection analyses

Sterile coverslips (12 mm diameter) were placed inside 12‐well plates (one per well). L‐cells in DMEM10 (4 × 10^3^ cells per well) were seeded over them and allowed to grow for 24 h [33, 34, 35]. At 48 h PI (day 5), glass coverslips were washed with PBS and fixed in 4% (paraformaldehyde) in 0.1% Triton X‐100‐PBS [33, 34, 35]. Infected cells were incubated with primary ab Tc‐cyp19 (1 : 1000) followed by incubation with secondary anti‐rabbit IgG Alexa fluor^®^ plus 488 (1 : 200) catalog #A32790 (Thermo Fisher Scientific, Waltham, MA, USA). Primary ab Tc‐cyp19 binds specifically to intracellular amastigotes. Next, cells were stained with (4′,6‐diamidino‐2‐phenylindole) (DAPI). Infection was determined microscopically by evaluating the percentage of infected cells containing intracellular amastigotes and counting no fewer than 300 cells per coverslip [33, 34, 35]. Infected cells per microscopic field were visualized using a fluorescence microscope (Nikon Microphot‐SA, Melville, NY, USA) at a magnification of 400×.

### Serum measurements

Serum concentration levels of GH and IGF‐1 were determined from each GHR^−/−^ or bGH mice and WT mice using mouse/rat Enzyme‐linked immunosorbent assay ELISA before infection. Commercial kits from ALPCO^®^ for mouse/rat‐growth hormone ELISA kit catalog number (22‐GHOMS‐E01) https://www.alpco.com/store/mouse‐rat‐growth‐hormone‐elisa.html and mouse/rat IGF‐1 ELISA catalog number (22‐IG1MS‐E01) https://www.alpco.com/store/mouse‐rat‐igf‐1‐elisa.html from New Hampshire (USA) were used following the manufacturer's ALPCO^®^ instructions. Cytokines levels from plasma collected from males (GHR^−/−^ and bGH with respective WT fasted for 12 h overnight) were measured using a Miliplex Mouse Cytokine/Chemokine Panel I (Cat. No. MCYTOMAG‐70K‐PX32) and Mouse Metabolic Hormone (Cat. No. MMHMAG‐44K). All Millipex panels were analyzed using a Milliplex 200 Analyzer (Millipore, Burlington, MA, USA) according to the manufacturer's instructions previously described [36].

### Bioinformatic analysis

Previously RNA‐Seq data [37, 38, 39] from human foreskin fibroblast cells infected with *T. cruzi* strain CL Brenner and Sylvio at 96 and 72 h postinfection (hpi) were selected to generate a heat map. Four differentially expressed genes (*GHR, IGF‐1, IGF‐1R, IGFBP3*) RNA‐seq data were extracted and plotted. r version 4.2.1 was used with rstudio version 2022.07.1 (Vienna, Austria) and the following packages to generate the heatmap: tidyverse 1.3.1, complexheatmap 2.13.0, and readxl 1.4.0.

### Statistical analysis

In total, GHR^−/−^ (*n* = 6), WT (*n* = 6), bGH (*n* = 6), and WT (*n* = 6) serum sample treatments were used for experiments. Results were expressed as mean ± standard error (SE). An unpaired *t*‐test was performed in graphpad prism version 9.1.2 (San Diego, CA, USA). A *P*‐value of < 0.05 was considered statistically significant.

## Results

### GH modulates *T. cruzi* infection via bioinformatic analysis and *in vitro*

Using previous RNA‐seq data [37, 38, 39] from differentially expressed genes, the heat map showed that GHR gene expression is downregulated in human foreskin fibroblast cells infected with *T. cruzi* (Fig. 1A). Importantly, these data indicate that different *T. cruzi* strains (Sylvio, CL Brenner), at different times of infections (96 and 72 hpi), consistently downregulate *GHR* expression. In normal conditions, *GHR* levels are downregulated as *T. cruzi* infection progresses. These data coincide with our previous *in vitro* studies that showed exogenous treatment of cells with high levels of GH decreased *T. cruzi* infection [24]. Together, these data imply that GH plays a modulatory role during *T. cruzi* infection and coincides with our hypothesis that high GH levels decreased *T. cruzi* infection [24]. We then simulated LS conditions *in vitro* and found that L‐cells infected with *T. cruzi* and treated with high GH concentrations levels (200 ng·mL) + low IGF‐1 (50 ng·mL) significantly decreased the number of the infected cells by 35% (*P* < 0.01) compared with 90% in control cells (2% FBS) (Fig. 1B–D). We also found that 10% FBS treatment significantly decreased the number of infected cells by 60% (*P* < 0.01) compared with 90% control (2% FBS), possibly by GH and growth factors included in the commercial 10% FBS composition (GH ~ 39 ng·mL, PRL ~ 176 ng·mL, IGF‐1 ~ 111 ng·mL, Insulin ~ 10 ng·mL). We did not find any significant changes in infection when L‐cells were treated with high GH levels (200 ng·mL) + high IGF‐1 levels (900 ng·mL) DMEM, simulating AC conditions *in vitro*.

### Serum from GHR^−/−^ mice decreases *T. cruzi* infection

Serum extracted from WT mice (*n* = 6) and GHR^−/−^ mice (*n* = 6) was quantified for GH and IGF‐1 concentrations immediately before treatment of the cells (Fig. 2A). In adult WT mice, the physiological serum levels for GH range between 0.2 and 11 ng·mL and for IGF‐1 between 400 and 800 ng·mL [40, 41, 42]. In our results, similar physiological serum levels were found [GH (0.33 ± 0.19 ng·mL) and IGF‐1 (675 ± 56 ng·mL)] in WT mice (Fig. 2B,C). As expected, GHR^−/−^ serum mice had extremely high GH (506 ± 49 ng·mL) and extremely low IGF‐1 levels (11 ± 7.12 ng·mL) (Fig. 2B,C). Cytokine levels from GHR^−/−^ mice showed significantly increased levels of granulocyte colony‐stimulating factor (G‐CSF) (681 pg·mL, *P* < 0.001) compared with controls (165.73 pg·mL) (Table 2). On the contrary, significantly decreased levels of interleukin‐1α (IL‐1α) (99.39 pg·mL, *P* < 0.007) compared with controls (180.69 pg·mL) were found (Table 2). Regarding infection, serum treatment from GHR^−/−^ mice significantly decreased the number of infected cells by one‐half (28 ± 3.9%, *P* < 0.01) compared with WT serum treatment (48 ± 4.63%) (Fig. 2D,E). Together these results show that treatment with elevated GH and low IGF‐1 serum collected from GHR^−/−^ mice decreases *T. cruzi* infection in mammalian cells.

**Table 2:** Cytokine levels from serum collected from WT and GHR^−/−^ mice. Values are given as mean ± SEM, and units are pg·mL plasma. ND, values could not be read or determined.

Cytokine	WT	GHR^−/−^	*P*‐value
G‐CSF	165.73 ±33.11	681.44 ± 121.51	0.001*
GM‐CSF	21.66 ± 9.03	20.36 ± 6.87	0.911
INF‐γ	ND	ND	–
IL‐1α	180.69 ± 20.28	99.39 ± 15.84	0.007*
IL‐1β	44.04 ± 36.36	11.91 ± 9.72	0.408
IL‐2	ND	ND	–
IL‐6	2.36 ± 1.56	3.42 ± 1.18	0.594
IL‐4	ND	ND	–
IL‐10	2.21 ± 1.30	1.59 ± 1.04	0.715
IL‐13	15.47 ± 10.79	5.05 ± 2.50	0.363
IL‐17	5.89 ± 1.21	3.19 ± 0.42	0.055
KC	33.57 ± 5.75	70.68 ± 12.83	0.019*
TNF‐α	14.17 ± 12.57	3.77 ± 3.77	0.441

* Significant ρ < 0.05 using *t*‐test.

### Serum from bGH mice decreases *T. cruzi* infection

Serum extracted from WT mice (*n* = 6) and bGH mice (*n* = 6) was quantified for GH and IGF‐1 concentrations on each mouse immediately before cell treatment (Fig. 3A). Physiological serum levels of GH (5 ± 2.8 ng·mL) and IGF‐1 (768 ± 18 ng·mL) were found in WT mice (Fig. 3B,C). As expected, bGH mice showed extremely high GH (> 2000 ng·mL) and IGF‐1 (1657 ± 112 ng·mL) serum levels (Fig. 3B,C). Cytokine levels from bGH mice showed significantly increased levels of interleukin 1β (IL‐1β) (32.29 pg·mL, *P* < 0.015) compared with controls (7.09 pg·mL), as well as significantly increased levels of interleukin‐13 (IL‐13) (32.64 pg·mL, *P* < 0.003) compared with controls (6.15 pg·mL) (Table 3). Regarding infection, bGH mouse serum treatment significantly decreased the number of infected cells (41 ± 1.8%, *P* < 0.04) compared with WT serum (54.1 ± 5.3%) (Fig. 3D,E). Together, these data suggest that elevated GH and + IGF‐1 serum levels reduce *T. cruzi* infection of mammalian cells but to a lower degree relative to the results obtained when GHR^−/−^ serum was used.

**Table 3:** Cytokine levels from serum collected from WT and bGH mice. Values are given as mean ± SEM, and units are pg·mL plasma. ND, values could not be read or determined.

Cytokine	WT	bGH	*P*‐value
G‐CSF	335.08 ± 59.00	336.35 ± 65.63	0.980
GM‐CSF	23.12 ± 4.83	35.79 ± 6.45	0.130
INF‐γ	3.39 ± 2.83	3.17 ± 2.62	0.954
IL‐1α	304.14 ± 81.06	149.40 ± 30.76	0.090
IL‐1β	7.09 ± 1.10	32.29 ± 9.13	0.015*
IL‐2	11.73 ± 11.72	0.68 ± 0.67	0.360
IL‐6	8.47 ± 5.49	13.25 ± 5.23	0.530
IL‐4	ND	ND	–
IL‐10	56.72 ± 45.86	3.22 ± 1.03	0.330
IL‐13	6.15 ± 1.48	32.64 ± 7.31	0.003*
IL‐17	5.41 ± 1.07	8.72 ± 6.01	0.040*
KC	50.48 ± 1.50	33.91 ± 5.08	0.030*
TNF‐α	1.66 ± 15.40	8.87 ± 5.71	0.220

* Significant ρ < 0.05 using *t*‐test.

## Discussion

This study is the first to characterize the effect of serum derived from GHR^−/−^ and bGH mice on *T. cruzi* infection. We observed a significant decrease in the number of infected cells after treatment with GHR^−/−^serum, providing further evidence of the role of GH during *T. cruzi* infection. These results agree with our previous *in vitro* studies showing that high GH levels and the combination of high GH and low IGF‐1 (LS conditions) decrease *T. cruzi* infection in mammalian cells.

In this *ex vivo* study, both mice lines have increased serum GH levels but differ in corresponding serum IGF‐1 concentrations. Treatment with GHR^−/−^ mice serum (with elevated GH and low IGF‐1) decreased *T. cruzi*‐infected cells by 28% compared with 48% in WT controls. By contrast, treatment with bGH mouse serum (with elevated GH and IGF‐1) decreased the number of infected cells by 4% compared with 54% in WT controls. Therefore, serum from mice with elevated GH and decreased IGF‐1 levels appear to have a more substantial effect on the parasite's ability to infect mammalian cells than serum with elevated GH and IGF‐1 levels. Thus, elevated IGF‐1 levels may partially alter the inhibitory effect of GH on infection. We are aware that the effect of decreasing *T. cruzi* infection with serum from each strain of mice could also be mediated by cytokines, proteases, and other proteins present in the GHR^−/−^ (increased G‐CSF) and bGH (increased IL‐1β) serum. For example, previous studies have shown that increased G‐CSF in mice has been reported to promote the development, mobilization, and activation of neutrophils, leading to protection against infections [43]. By contrast, IL‐1β is known to play a role in coordinating host immune and proinflammatory responses [44]. In our results, serum cytokines showed only a few differences between both mice models (increased TNF‐α in GHR^−/−^ and decreased levels in bGH mice compared with WT controls); thus, further *in vivo* experiments are needed to clarify this matter.

Although this is the first *ex vivo* study using LS mice (GHR^−/−^) to characterize the potential effects of GH on *T. cruzi* infection, these findings are concordant with previous *in vitro* and *in vivo* studies that indicate *T. cruzi* infection may lead to hypothalamic–pituitary–adrenal axis (HPA) imbalance (Table 1) [18]. *In vivo* mice models have also shown that during *T. cruzi* infection, modulation of pituitary hormones PRL and GH and adrenal glucocorticoids (GC) caused immune suppression and thymic atrophy by CD4^+^CD8^+^ T‐cell depletion. Moreover, data from previous RNA‐Seq analysis [37, 38, 39] show that GHR gene expression is consistently downregulated during *T. cruzi* infection (Fig. 1A). These data indicate that GHR levels are altered as *T. cruzi* infection progresses, implying that the GH/IGF‐1 axis might also play a detrimental role during infection. In support of these findings, our *ex vivo* results show that high GH levels in circulation are associated with protection against *T. cruzi* infection in mammalian cells.

Of note, GHR^−/−^ mice have elevated serum GH and decreased IGF‐1 and, despite being short and obese, display low serum insulin concentrations along with improved insulin sensitivity. By contrast, bGH mice with excess serum GH and IGF‐1 levels have decreased adiposity, insulin resistance, and high susceptibility to diabetes [26]. The very high GH serum levels found in both mouse lines appear to protect against *T. cruzi* infection. Our findings correlate with the clinical observation that no clinical cases of *T. cruzi* infection were reported in Ecuadorian LS subjects. Remarkably, LS subjects are obese, display high insulin sensitivity, and have diminished incidence of cancer and insulin‐resistant diabetes [14]. The absence of the GH counter‐regulatory effects of GH on carbohydrate metabolism, despite the very high serum GH levels, as well as the low serum IGF‐1 and insulin levels documented in LS subjects, have been proposed to explain the diminished incidence of these diseases [13, 14]. Our previous *in vitro* findings and the present observations in this *ex vivo* report suggest that high circulating GH and low circulating IGF‐1 levels might be, at the very least, partially protecting LS subjects from *T. cruzi* infection.

Interestingly, in a study from Barrios et al. [45], when isolated GHD macrophages from patients from Brazil [17] were treated with IGF‐1 *in vitro*, there was an increased infection with the parasite *Leishmania* spp. (closely related to *T. cruzi*). These data correlate with our previous *in vitro* study, where high levels of IGF‐1 also increased *T. cruzi* infection *in vitro* [24]. Additionally, the study of GHD patients from Brazil showed that GH deficiency is not associated with an increased frequency of infectious diseases such as CD, Leishmaniasis, HIV, hepatitis B, and C compared with controls [17]. Thus, altered GH action, as seen in our LS model, seems to play a protective role during infectious diseases that need further exploration.

In summary, we report decreased *T. cruzi in vitro* infection in the presence of serum collected from two modified mouse lines (GHR^−/−^ and bGH) with altered GH action. Even though a direct and indirect influence of *T. cruzi* in endocrine homeostasis through HPA axis imbalance has been documented, the relationship between LS patients and resistance to *T. cruzi* infection has only recently been explored [24]. Our results suggest that the high circulating GH serum levels may confer partial protection against *T. cruzi* infection in humans. These data are consistent with our previous *in vitro* findings showing that high serum GH levels, as seen in LS patients, confer resistance to *T. cruzi* infection. This study also highlights the potential of using GH to decrease infectivity, an event worth considering when treating patients during the acute and chronic CD phases. Although additional studies are needed to fully understand the direct or indirect mechanisms of GH action during *T. cruzi* infection, our findings provide a potential mechanism for explaining the absence of clinical *T. cruzi* infection observed in LS individuals.

## Conflict of interest

The authors declare no conflict of interest.

### Peer review

The peer review history for this article is available at https://www.webofscience.com/api/gateway/wos/peer‐review/10.1002/2211‐5463.13627.

## Author contributions

PM‐C, RB, and JJK involved in conception and design of the work. PM‐C, YQ, and JAC involved in data analysis and interpretation. KF, SB, and PM‐C contributed to sample collection. JAY contributed to bioinformatic analysis. EOL contributed to cytokines analysis. JJK, RB, MJG, and JG‐A involved in critical article revision. JJK and JG‐A involved in final approval of the version to be published.

## Data Availability

The data that support the findings of this study are available from the corresponding author (kopchick@ohio.edu) upon reasonable request.
